# NOT KNOWING OTHER AFRICAN AMERICAN BEREAVED DEMENTIA CAREGIVERS: SEMISTRUCTURED INTERVIEW RESULTS

**DOI:** 10.1093/geroni/igad104.0030

**Published:** 2023-12-21

**Authors:** Ashley Millenbah, Robbin Frazier, Sheryl Fairbanks, Warren Wolfe, Zachary Baker

**Affiliations:** University of Minnesota, Minneapolis, Minnesota, United States; University of Minnesota, Center for Healthy Aging and Innovation, Minneapolis, Minnesota, United States; University of Minnesota, Minneapolis, Minnesota, United States; Freelance Journalist, Roseville, Minnesota, United States; Arizona State University, Tempe, Arizona, United States

## Abstract

Semi-structured interviews focused on African American bereaved dementia caregivers and the individual who may serve them in the Twin Cities of Minnesota. The four questions that drove these interviews were linear in nature: 1. What needs exist among African American bereaved dementia caregivers in the Twin Cities? 2. What services exist to help these individuals meet their needs? 3. If unmet needs exist, would the group support model meet those needs? and, 4. If this model is promising, what adaptations might it need to best serve African American bereaved dementia caregivers? Interviews showed that needs exist at points before and after caregiving ends to increase education and destigmatize dementia in African American communities to facilitate understanding, acceptance, and validation during and after caregiving. Support from those who have gone through similar situations is increasingly important both during and after dementia caregiving, but identifying those in similar situations is difficult (few could identify any other African American Bereaved Dementia Caregivers besides themselves). While a culturally specific support group model was of interest to some to create a feeling of comfort, others emphasized that the main importance was a bonding over shared experiences, regardless of cultural background. A former dementia caregiver support group might be held in various locations and hosted by varying individuals, including at a church, hair salon, or even a grocery store. Overall, it was seen as important for there to be a specific meaning, purpose, and goals for a group of African American former dementia caregivers.

